# Longitudinal study of adolescent tobacco use and tobacco control policies in India

**DOI:** 10.1186/s12889-018-5727-8

**Published:** 2018-07-03

**Authors:** Ritesh Mistry, Mangesh S. Pednekar, Prakash C. Gupta, Trivellore E. Raghunathan, Surekha Appikatla, Namrata Puntambekar, Keyuri Adhikari, Maqsood Siddiqi, William J. McCarthy

**Affiliations:** 1 Department of Health Behavior and Health Education, University of Michigan School of Public Health, 1415 Washington Heights, SPH I, Room 3806, Ann Arbor, MI 48109-2029 USA; 2 Healis Sekhsaria Institute for Public Health, Navi Mumbai, India; 3 Department of Biostatistics, University of Michigan, Ann Arbor, USA; 4 Survey Research Center, Institute for Social Research, University of Michigan, Ann Arbor, USA; 5 Cancer Foundation of India, Kolkata, India; 6 Department of Health Policy and Management, University of California, Los Angeles, USA

**Keywords:** Tobacco, Adolescents, Policy, Compliance

## Abstract

**Background:**

This project will use a multilevel longitudinal cohort study design to assess whether changes in Community Tobacco Environmental (CTE) factors, measured as community compliance with tobacco control policies and community density of tobacco vendors and tobacco advertisements, are associated with adolescent tobacco use in urban India. India’s tobacco control policies regulate secondhand smoke exposure, access to tobacco products and exposure to tobacco marketing. Research data about the association between community level compliance with tobacco control policies and youth tobacco use are largely unavailable, and are needed to inform policy enforcement, implementation and development.

**Methods:**

The geographic scope will include Mumbai and Kolkata, India. The study protocol calls for an annual comprehensive longitudinal population-based tobacco use risk and protective factors survey in a cohort of 1820 adolescents ages 12–14 years (and their parent) from baseline (Wave 1) to 36-month follow-up (Wave 4). Geographic Information Systems data collection will be used to map tobacco vendors, tobacco advertisements, availability of e-cigarettes, COTPA defined public places, and compliance with tobacco sale, point-of-sale and smoke-free laws. Finally, we will estimate the longitudinal associations between CTE factors and adolescent tobacco use, and assess whether the associations are moderated by family level factors, and mediated by individual level factors.

**Discussion:**

India experiences a high burden of disease and mortality from tobacco use. To address this burden, significant long-term prevention and control activities need to include the joint impact of policy, community and family factors on adolescent tobacco use onset. The findings from this study can be used to guide the development and implementation of future tobacco control policy designed to minimize adolescent tobacco use.

## Background

Tobacco use prevention and control are public health priorities globally and in India. The prevalence of adult current tobacco use in India is 42% for males and 14% for females [1], and adolescent current tobacco use prevalence is 19% for males and 8% for females [2]. Annually, nearly 1.3 million people die in India from a tobacco-related disease [3, 4]. Although tobacco control policies are important strategies to reduce population level tobacco use, the extent and role of partial compliance are not well understood [5, 6]. A key set of India’s tobacco control policies pertain to Articles 8, 13 and 16 of the FCTC (WHO Framework Convention for Tobacco Control) regarding secondhand smoke exposure, access to tobacco products and tobacco promotion, respectively. Major provisions of national and local laws regulate where tobacco can be used, sold and advertised (e.g., Sections 4–7 of the Cigarettes and Other Tobacco Products Act or COTPA). Reports from several urban areas in India suggest that compliance was consistently low for restrictions on tobacco advertisements at the point-of-sale [6, 7], and was moderate for bans on the sale and marketing of tobacco near schools in Mumbai [8] and Ahmedabad [9], high for smoke-free laws in one district in Punjab [10], and high for the ban on the sale of *gutkha* (a flavored smokeless tobacco product) [11, 12]. The study described in this paper will gather and analyze population-based, prospective data on compliance with existing tobacco control laws in Mumbai and Kolkata, India. The primary aim is to assess how variations in compliance with tobacco point-of-sale and smoke-free laws are associated with tobacco use in adolescents.

### Community tobacco environment

We define the Community Tobacco Environment (CTE) as places within community neighborhoods where tobacco products are sold, advertised and used, and level of compliance with existing point-of-sale and smoke free laws. Observational studies suggest that reducing the number of places where tobacco products are sold [13–17] and advertised, especially near schools, may be effective in reducing youth tobacco use [18, 19], including in India [8, 20–22]. With some exceptions [15, 19], past studies were limited to using cross-sectional data and indirect environmental measures, e.g., self-reports about community exposures to tobacco advertisements [20]. The retailer point-of-sale (POS) environment is being increasingly recognized as an important setting in which policies may be used to reduce the purchase and consumption of tobacco and other health damaging products by adolescents [23]. Exposure to tobacco advertisements and products at the point-of-sale has been linked to adolescent tobacco use [19], increased brand recognition in students [24], impulse purchases of tobacco in smokers [25], and exposures are concentrated in low-income neighborhoods [26]. Laws that ban smoking in public places are associated with lower smoking in youth [27, 28].

### Family influences

The influence of the CTE on adolescent tobacco use may be moderated by family contexts*.* The family environment has important influences on adolescent development and resiliency factors [29, 30] that can mitigate against risks in the environment. For example, demanding and responsive parenting styles have been shown to bolster adolescent resiliency [31], and reduce adolescent health risk behaviors [32, 33], including tobacco use [33–35]. Though research from India identifies risks associated with tobacco use by family members [36], to our knowledge there is a dearth of published research on how parenting styles and practices in India may be associated with adolescent tobacco use, and how parenting interacts with environmental influences.

### Individual level factors

Individual level factors may partially mediate the relation between CTE factors and adolescent tobacco use. For instance, perceived ease of access to tobacco [37], perceived norms about tobacco use [38], and perceived exposure to tobacco advertisements in the community have been linked to youth tobacco use. These factors may help explain associations between adolescent tobacco use and CTE factors. High youth receptivity to tobacco-promoting messages and exposure to advertisements is an ongoing challenge for tobacco control because they have been shown to increase risk of youth tobacco use in India [20].

### Conceptual framework

Multilevel approaches that incorporate community, family and individual level factors can provide a richer understanding of the impact of tobacco control policies on adolescent tobacco use [39–41]. With some exceptions [40, 42], research has primarily focused on individual and family environmental factors, or individual and community environmental factors, but more research is needed that include the contribution of community, family and individual level factors *simultaneously* when assessing the multilevel determinants of adolescent tobacco use behaviors [43, 44].

**Fig. 1:**
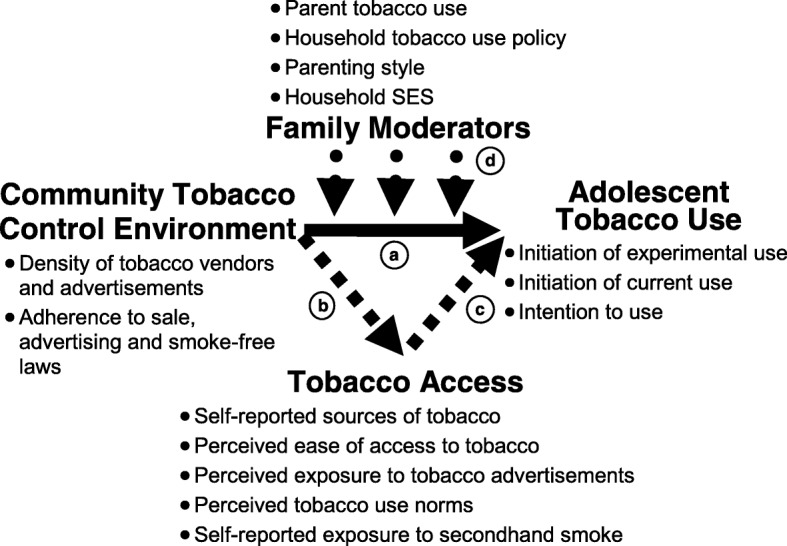
Conceptual Framework

Our multilevel framework is guided by a socio-ecological perspective [45] that draws from Social Cognitive Theory [46], the Theory of Triadic Influences [47] and resiliency theory [31, 48]. Social Cognitive Theory posits reciprocal causation between health behaviors, cognition/affect and environmental factors. Theory of Triadic Influences states that behaviors are determined by broader social economic factors, immediate social contexts (e.g. family), and individual characteristics. Resiliency theory provides a positive youth development perspective that focuses on internal and external assets (mental health, family support, good parenting, peers who don’t use) that mitigate the effect of risk factors. The framework includes community context (e.g. CTE factors), the immediate social context (i.e., family context and peer influences), and individual level factors such as knowledge, perceptions and affect. Figure 1 shows the hypothesized associations between the CTE factors and adolescent tobacco use (arrow a). Factors measuring adolescent access to tobacco products are hypothesized partial mediators (arrows b and c). Family factors are hypothesized moderators (arrow d) that protect against or enhance community level effects (arrow e). For example, parent support may buffer the effect of high tobacco vendor density on adolescent tobacco use, and parent tobacco use may exacerbate this effect.

### Research questions

The project will address the following research questions and hypotheses:

- What is the association between adolescent tobacco use initiation and compliance with tobacco control laws about tobacco sales to minors, advertisements and smoking in public places? *Hypothesis*: Adolescent tobacco use initiation will be negatively associated with community compliance with COTPA tobacco control laws.
- What is the association between adolescent tobacco use initiation and the density of tobacco vendors and tobacco advertisements? *Hypothesis*: Adolescent tobacco use initiation will be positively associated with greater density of tobacco vendors and tobacco advertisements.
- Does adolescent perceived access to tobacco products and perceived norms about tobacco use partially mediate the association between CTE factors and adolescent tobacco use initiation? *Hypothesis*: Compared to non-users, adolescents who initiate tobacco use over the follow-up period will be more likely to report easier access to tobacco products and greater perceived prevalence of tobacco use, thus explaining some of the observed association of adolescent tobacco use initiation with variation in CTE factors.
- How do family factors modify the effect of CTE factors and adolescent tobacco use? *Hypothesis*: Parenting style, family conflict, parent tobacco use and household tobacco use policy will moderate the relationship between CTE factors and tobacco use initiation.

## Methods

We will conduct a comprehensive longitudinal population-based household survey of 1820 adolescents 12–14 years of age and their parent/main caregiver, with data collection occurring at baseline (Wave 1), 12-month (Wave 2), 24-month (Wave 3) and 36-month follow-ups (Wave 4). The project will be conducted in Mumbai and Kolkata, India; two large, populous, geographically dispersed urban areas that reflect India’s urban variation in the prevalence of tobacco use, tobacco control policy implementation, socioeconomic development, infrastructure and cultural factors.

### Sampling plan

A multi-stage sampling design will be used to obtain a representative sample of communities and adolescents in both cities. We will use a comprehensive sampling frame from the National Sample Survey Organization (NSSO) of the Ministry of Statistics and Programme Implementation called the Urban Frame Survey [49], which covers all populated urban areas within the country. The geographies of the sampling frame are hierarchically nested: States, Cities, Investigator Units (IV units) and Blocks. We will select Mumbai, Maharashtra and Kolkata, West Bengal for our sampling frame and include Blocks designated by the NSSO as Affluent Areas, Residential Areas and Slum Areas [49]. These areas represent 97% of all Blocks in both cities. We will sample 11 IV units per city and then 4 Blocks on average per IV unit for a total of 88 Blocks (2 cities × 11 IV Units × 4 Blocks per IV unit). All eligible households within sampled Blocks will be approached for enrollment in the study. If the target sample size is not achieved from the 88 sampled Blocks, additional blocks will be sampled as needed. Eligible households will be defined as those having at least one adolescent aged 12–14 years living with his/her primary adult caregiver. Adolescent sampling weights will be developed using base weights proportional to the reciprocal of the product of the selection probabilities at each stage with adjustment for unit non-response.

### Data collection activities

#### Household component

Household data collection will include interviewer-administered adolescent and parent/primary caregiver surveys. The adolescent survey will be adapted from our past research [6, 8, 14, 50–52], and guided by our conceptual framework and the literature on the most important risk and protective factors for tobacco use. It will include items found in the Global Youth Tobacco Survey-India [2], Mumbai Student Tobacco Survey [6, 8], and the Tobacco Control Policy – India survey, an adaptation of the International Tobacco Control Policy Evaluation Project Survey [53]. We will emphasize policy components such as bans on the sale of gutkha, smoke-free policies, and policies restricting tobacco advertisements*.* We will also include items about interactions with tobacco retail environments in home and school neighborhoods as well as COTPA defined public places. The survey will include measures of smoking and smokeless tobacco use (including gutkha, hookah and e-cigarettes), tobacco use risk factors and family factors. The parent survey will be guided by the Global Adult Tobacco Survey [54] and the literature about family risk and protective factors for adolescent tobacco use. The survey interviews will be conducted English, Hindi, Marathi and Bengali by trained field investigators using a computer-aided personal interviewing system.

#### Community component

We will measure CTE factors by collecting GIS data about all tobacco vendors and tobacco advertisements in the 22 sampled IV units. We will conduct audits of all tobacco POS environments and COTPA defined public places to measure compliance with POS and smoke-free laws. Baseline GIS data will be collected during Wave 1, and will be updated at Wave 4.

##### Mapping

In each community, all tobacco-related community features will be mapped. A “community” will be defined as the IV unit in which the sampled household is located. GPS enabled tablet computers will be used to geocode locations of all tobacco vendors, tobacco advertisements, COTPA defined public places (hospital buildings; restaurants; public offices; court buildings, educational institutions; bus stops). The mapping activities will be conducted by field teams trained to use the field GIS mapping equipment and procedures. Detailed mapping and training protocols will be adapted from our previous experience [8] and the literature [55, 56].

##### Tobacco POS audits

All tobacco vendors will be audited for compliance to POS laws. We will adapt from our previous experience [6] and the existing literature [57]. The audits will focus on compliance with tobacco POS provisions. POS audits that we used previously [6] had compliance items with good internal consistency (α = 0.78), face validity and construct validity. Compliance will be measured (Yes = 1; No = 0) for each POS provision **(**Table 1**)**, and summed to create a POS Compliance Score. In a sample of mapped tobacco vendors, we will measure compliance with the ban on tobacco sales to minors by using trained actors who look like minors. Successful purchase attempts will be recorded (Succeed = 1; Not succeed = 0). The procedures will be guided by the literature on conducting compliance checks to tobacco sale to minors [58–60].

**Table 1:** Tobacco point-of-sale audit

Is not located within 100 yards of educational institutions	
Presence of signage about the ban on tobacco sales to minors	
Presence of two or fewer tobacco advertisements	
Tobacco advertisement meets size, content and warning specifications	
Tobacco packages have required textual and pictorial warnings	
Did not sell tobacco to minors	
Did not sell gutkha sales	

##### Public place audits

All COTPA defined public places will be audited for compliance with smoke-free laws. We will adapt existing methods [61], and those recommended by researchers in India [62, 63]. Presence/absence (Yes = 1; No = 0) of items in Table 2 will be noted, and observations will be summed to create a Smoke-free Compliance Score.

**Table 2:** Public place smoke-free audit

Presence of no smoking signage	
Absence of active smoking	
Absence of smoking aids, e.g., ashtrays, matchboxes, lighters	
Absence of cigarettes butts or bidi ends	
Responses by patrons saying they did not observe anyone smoke	

#### Measures

*Primary outcomes* The *primary outcomes* will be initiation of experimental tobacco use and initiation of current tobacco use between Wave 1 (baseline) and Wave 4 surveys using existing methods [64, 65], and adapting them for smoking and smokeless tobacco products in India. Initiation of experimental use at follow-up will be measured as reports of trying any tobacco product for the first time during the last 12-months. Initiation of current use will be measured as reports of tobacco use within the 30 days prior to the survey and having initiated experimental use prior to that 30-day period. The *secondary outcome* will be tobacco use intention, a consistent predictor of subsequent use in never users [66]. Finally, tobacco use initiation at baseline will be measured by asking ever users about age of initiation.

##### Community tobacco environment

Data from GIS mapping, Tobacco POS Audits and Public Place Audits will be used to measure the following community factors:

- *Tobacco vendor and tobacco advertisement density:* Spatial data about tobacco vendors and tobacco advertisements will be used to measure vendor density, proximity and clustering based on prior work [8, 14, 15, 67, 68]. We will use several measures of spatial distribution including density, clustering (hotspots), and distance [69]. Density will be calculated as the ratio of enumerated features (i.e., vendors) to square meters and to population size in the overall community. We will also calculate location quotients to measures the concentration of tobacco vendors and tobacco advertisements [70]. Clustering will be measured through nearest neighbor analysis [70], spatial autocorrelation using the Global Moran’s I statistic [71], and hotspot/cold spot analysis using the Getis-Ord Gi* Statistic [72].
- *Community compliance with POS policies and smoke-free policies:* For each community, the POS Compliance Score and Smoke-free Compliance Score described above will be averaged across all sampled tobacco vendors to create the Community POS Compliance Score and Community Smoke-free Compliance Score.

##### Mediators and moderators

To identify pathways by which CTE factors are associated with tobacco use, the following partial mediators will be examined: self-reported sources of tobacco, perceived ease of access, exposure to tobacco advertisements, perceived tobacco use norms and self-reported exposure to secondhand smoke. Moderators will include parental tobacco use, parenting style, parental support, household tobacco use policy and household SES.

##### Potential confounders

To address confounding, we will include factors guided by our conceptual framework and the literature [19, 41, 73–76] about community (SES, distribution of religions, population density), family (parent-child communication about tobacco) and adolescent level factors (religion, age, gender, peer tobacco use, depressiveness).

All data will be stored on secured and password-protected computers, and only IRB certified study team members will have access to the data. Personal identifiers will be stored for follow-up data collection. A data quality assurance plan outlines the quality standards and reporting requirements.

#### Statistical analysis

We will assess whether CTE factors are associated with adolescent tobacco use initiation, and measure the role of moderators and partial mediators (Fig. 1). Since individuals will be nested within communities, data analysis will be conducted using a multilevel random-effects regression framework [77, 78] using Stata and GLLAMM [79].

##### Direct associations

Model building will be sequential, starting with a model for the outcome including CTE factors then adding family and individual level factors in blocks to examine whether associations hold statistically after inclusion of variables at each level. In addition to Wald tests of the significance of single predictors, likelihood ratio tests will be used to assess the joint significance of sets of related predictors (e.g., multiple vendor density and compliance measures). In longitudinal analyses, we will also estimate growth curves [80] for community level compliance latent classes.

*Multilevel moderation analysis* will assess whether the family factors are moderators. We will use cross-level analysis to assess moderation by testing interaction terms between level 1 and level 2 variables [81], e.g., between community compliance and family context (shown as arrow d in Fig. 1**)**.

##### Mediation analysis

Mediation (See Fig. 1, see arrows a, b and c) will be assessed in several ways. Statistically significant associations for arrows a, b and c will indicate partial mediation [82, 83]. We will also use structural equation modeling to assess multiple mediators simultaneously [84].

##### Statistical power

Our power calculations account for the ​clustering of households in the sample ​design, and assume ​a range of ​community intraclass correlations (ICC) of 0.01–0.05 [8]. Based on a 85% retention, we expect an effective sample size to be between 1199 (design effect of 1.29 or ICC of 0.01) and 631 (design effect of 2.45 or ICC of 0.05). Power calculations were performed for two scenarios. First, to assess the effect of community level variables on the individual level variables,​ the random intercept​ logistic regression​ model (each community having a different intercept) with initiation of tobacco use as the outcome variable and a CTE variable at the community level as the primary predictor ​was used [85]. This analysis showed that we have > 80% power to detect standardized regression coefficients of sizes 0.07 to 0.09. Second, we conducted the power analysis for detecting the interaction effects between community level variables and individual level covariate (such as family factors) on adolescent level outcomes. Here the detectable (with 80% power) standardized regression coefficient for the interaction effects ranged between 0.11 and 0.15 [86]. Sample sizes on the order of 600 are adequate to detect small mediational effects with 80% power [87]. Hence, we expect our sample to provide adequate power for planned analyses.

## Discussion

India is well positioned to address its tobacco use problem because of a strong foundation of existing national, state and local tobacco control policies. Youth are important beneficiaries of these laws because they are susceptible to tobacco access, exposure and marketing [13–17], which are widespread in urban India [8, 20–22]. Detailed policy implementation and compliance data from local communities where important tobacco control policies, tobacco products and marketing ultimately interface with community members, including youth, is badly needed. This study will contribute substantially to research on tobacco control policy implementation and the influence of policy compliance on adolescent tobacco use, a behavioral risk factor of immense public health importance in India and globally.

### Strengths and limitations

There are notable strengths and limitations to this study. First, we will obtain a representative sample of communities and adolescents in both cities, which will enhance generalizability to these major Indian cities, and to other urban areas in India and the region. Second, this study will recruit a longitudinal cohort to study adolescent tobacco use initiation and trajectories. An important challenge to validity is attrition, which can typically be 20% per year in US-based research [88]. We have obtained high retention in prior longitudinal studies in India of > 5.5 years [89, 90]. We will obtain contact information of all participants and relatives during Wave 1 to facilitate subsequent data collection.

The findings from this study are expected to be useful in guiding future tobacco control policy enforcement, development and implementation to reduce adolescent tobacco use in India. They can be generalized with some caution to other countries in the region, and perhaps even to communities consisting of recent Indian diaspora.

## Abbreviations

COTPA: Cigarettes and Other Tobacco Products Act
CTE: Community Tobacco Environment
GIS: Geographic Information Systems
GLLAMM: Generalized Linear Latent And Mixed Models
GPS: Global Positioning System
ICC: Intraclass correlation
IV Unit: Investigator Unit
NSSO: National Sample Survey Office
POS: Point-of-sale
WHO: World Health Organization
